# Recent Advances in treatment of urethral stricture disease in men

**DOI:** 10.12688/f1000research.21957.1

**Published:** 2020-05-05

**Authors:** Naside Mangir, Christopher Chapple

**Affiliations:** 1 Department of Functional and Reconstructive Urology, Royal Hallamshire Hospital, Glossop Road, Sheffield, S10 2JF, UK

**Keywords:** urethral stricture, urethroplasty, urethral reconstruction, regenerative medicine, tissue engineering

## Abstract

Urethral stricturing is a narrowing of the urethral lumen as a result of ischaemic spongiofibrosis. The main challenge of currently available treatment options is recurrence of the stricture. Recent advancements in the treatment of urethral strictures mainly came from the fields of regenerative medicine and tissue engineering. Research efforts have primarily focused on decreasing the recurrence of stricture after internal urethrotomy and constructing tissue-engineered urethral substitutes to improve clinical outcomes of urethroplasty surgeries. The aim of this article is to review the most recent advancements in the management of urethral stricture disease in men.

## Introduction

Urethral stricturing is a narrowing of the urethral lumen as a result of ischaemic spongiofibrosis ^1^. Several aetiologic factors have been proposed, including trauma due to urethral instrumentation, infection, and inflammatory disorders such as balanitis xerotica obliterans. These create the initial epithelial injury, which then heals by fibrosis, resulting in a reduction in the urethral caliber and impairment to the flow of urine. Urethral strictures (US) can have significant adverse effects on physical and psychosocial well-being ^2^.

The approach to US disease is mainly driven by the anatomical location of the stricture, whether it is in the anterior or posterior urethra, and its relationship to the distal urethral sphincter mechanism. Posterior US often result from direct trauma or surgical interventions, such as transurethral prostate resection or radical prostatectomy, and can be treated effectively with dilatation if they are a stenosis of the sphincetric area. Disruption injuries of the urethra, although causing a stenosis, are not true strictures as defined above because there is discontinuity of the urethra, for example following a pelvic fracture or in the bulbar urethra following a fall astride injury, which is an indication for excision primary anastomosis (EPA) of the damaged segment with anastomosis of the two healthy urethral ends. Short strictures affecting the proximal portion of the anterior urethra can also be treated by dilatation, endoscopic incision, or EPA, with success rates in excess of 90% in many series ^3, 4^. Longer strictures or strictures affecting more distal portions of the anterior urethra require substitution urethroplasty using tissue flaps or grafts. A variety of tissue grafts such as from the bladder ^5^ and colonic mucosa ^6^ have been used. Currently, there appears to be increasing support for the use of buccal mucosa grafts for substitution urethroplasty ^1^, which, based on current knowledge, should be considered to be the most appropriate material.

Direct vision internal urethrotomy (DVIU) is the first-line treatment for US in men; however, stricture recurrence is still a problem, with a stricture-free rate of around 20% in long-term follow-up ^7^. Current guidelines recommend that patients with longer (>2 cm in length), multiple, penile, or distal strictures and extensive periurethral spongiofibrosis should not be offered repeated DVIUs, as they have a very limited chance of a durable cure. The same applies to shorter strictures that have failed to respond to one urethrotomy or if the stricture recurs within 3 months of the first incision ^8^. Such patients should be offered a urethroplasty, which has success rates of up to 95% ^3^, with anastomotic repairs typically being more successful compared to augmented repairs, where success rates of 80–85% are commonly reported ^4^.

New advances in the treatment of US in males have mainly come from the fields of regenerative medicine and tissue engineering. This research has primarily focused on decreasing the recurrence of US after DVIU using regenerative therapies and constructing tissue-engineered urethral substitutes to improve clinical outcomes of urethroplasty surgeries. The aim of this article is to review the most recent advances in the management of US disease in men.

## Regenerative therapies to improve the outcomes of DVIU

The pathophysiologic processes leading to the formation of a US are still not completely elucidated. An initial epithelial injury followed by an abnormal wound healing process that leads to the formation of a progressive fibrotic scar is probably complex and multifactorial at a cellular and molecular level ^9^. DVIU works by cutting a scar through the diseased area and allowing the healthy spongy tissue to regenerate through secondary healing.

Fibrotic tissue formation is the final common pathway for many fibrotic diseases. Following on from this, intralesional injection of antifibrotic agents/drugs (e.g. mitomycin C, corticosteroids, etc.) after DVIU has been studied in the past ^10^. A summary of the current strategies underlying the current advances in the treatment of US disease is presented in Table 1. To date, there is limited evidence from small clinical trials to show that such an approach is effective in preventing the recurrence of US after DVIU.

**Table 1.** A summary of novel technologies in the treatment of urethral stricture disease.

	Proposed mechanism of action	Comment
**Strategies to improve the** **outcomes of endoscopic** **interventions**		
Intralesional injection of antifibrotic agents/drugs after direct vision internal urethrotomy (DVIU)	Tissue regeneration in the corpus spongiosum improved after the fibrotic scar is cut	Small clinical trials showed limited efficacy in preventing recurrence of stricture
Balloon dilatation of the stricture using drug-coated balloon catheters	Drugs released during balloon dilatation limit hyperactive cell proliferation and fibrotic scar formation at the urethral scar site	Short-term follow-up demonstrated safe use with good efficacy Long-term results from randomised controlled trials are required
Submucosal injection of cell- based regenerative therapy products after DVIU	Uses the anti-fibrotic and regenerative properties of platelet-rich plasma and mesenchymal stem cells	No major safety concerns with autologous cells; however, their efficacy is debatable mainly owing to the variability in the quality and quantity of cell therapy products
**Tissue engineering strategies** **to replace damaged urethra**		
Epithelial cell-seeded graft implantation	Mature cells, generally from buccal mucosa, are combined with injectable scaffolds that cells can attach to and proliferate from after endoscopically injected to the site of urethral damage	The main advantage comes from the concentration of therapy products at the site of injection Can be considered an endoscopic means of urethral reconstruction; however, not yet tested in clinical trials
Construction of tissue engineered urethral tissue for urethral reconstruction	This represents the state-of-the-art construction of artificial urethral tissue from cells isolated from biopsy samples and scaffolds from natural or artificial sources	Studied extensively in clinical trials with promising results The regulatory approval processes that need to be followed and the costs of such therapies can be a potential limitation when widespread clinical use is desired

A recent development in this area is the commercialisation of a drug-coated balloon catheter (Optilume™), which combines a balloon dilation technique and drug delivery. The highly lipophilic drug, paclitaxel, is released after balloon dilatation, limiting hyperactive cell proliferation and fibrotic scar formation ^11^. Paclitaxel is an antineoplastic drug that inhibits cell replication by stabilising intracellular microtubules. Paclitaxel is thought to inhibit the proliferation of ureteral smooth muscle cells and urothelial cells. The distribution of paclitaxel in the urothelial, submucosal, and smooth muscle layers has previously been demonstrated in a porcine model after ureteral dilatation, showing reduced inflammation with drug-eluting balloons ^12^. The pseudostratified epithelium of the urethral mucosa has also been shown to permit the distribution of paclitaxel in the muscular layer in a rabbit model ^13^. Nevertheless, there is no direct evidence to suggest an effect of paclitaxel on US. This technology is now undergoing clinical trials. The interim results of the first non-randomised clinical trial with 53 patients showed that such an approach could be used safely in the treatment of short bulbar US with an anatomical success rate of 70% at 12 months ^14^. The efficacy and safety will need to be studied in long-term randomised controlled clinical trials.

## Cell-based regenerative therapies

In addition to drug treatments, recently, cellular and non-cellular regenerative medicine products have been tried. A recent randomised controlled clinical trial investigated the submucosal injection of platelet-rich plasma (PRP) after DVIU and showed a reduction in the recurrence of stricture in patients with primary, short, bulbar strictures where stricture recurrence was confirmed by postoperative urethrography ^15^. PRP is a concentrated suspension of platelets that is obtained easily by centrifugation of whole blood with a separator to remove other cellular components. It has been shown to have a high concentration of biologically active proteins such as transforming growth factor (TGF)-β, platelet-derived growth factor, and vascular endothelial growth factor, which can promote angiogenesis, wound healing, and deposition of matrix proteins. The regenerative properties of PRP have been supported by *in vitro* and *in vivo* studies, and PRP injection is becoming increasingly popular for clinical application in the treatment of burns, wound healing problems, and orthopaedic soft tissue injuries ^16, 17^. Although autologous PRP would not be expected to be associated with any safety concerns, its efficacy is still debatable. In our view, this approach remains investigational, as there appears to be a significant lack of standardisation in terminology, content, and quality of the PRP used in clinical trials ^18^.

There have also been studies investigating the efficacy of mesenchymal stem cells (MSCs) in the treatment of US in men. MSCs are known to have a rich secretome that has a positive influence on tissue regeneration ^19^. In the context of US disease, adipose-derived MSCs have been shown to decrease collagen I and III deposition ^20^ and result in less-extensive urethral fibrotic changes in imaging and histology in rat models ^21^. In this model, a stricture was created by injection of TGF-β1 into the rat urethra; TGF-β is the main regulator of tissue fibrosis in many biological processes ^22^. In another study, TNF-α-induced exosomal miR-146a, an anti-inflammatory miRNA expressed in the MSC exosome, was shown to mediate the anti-fibrotic action of MSCs, counteracting stricture formation ^23^. Hence, there is some evidence from animal studies suggesting MSC injection could be beneficial as an adjunct to DVIU, but adequate clinical studies have yet to be performed.

MSCs have also been suggested as an adjuvant cellular therapy after substitution urethroplasty. In this context, bone marrow-derived MSCs have been shown to modulate the immune response with a significant reduction in pro-inflammatory cytokines TNF-α and IL-1β, a shift in collagen type from III to I, and wound healing in a rat model of substitution urethroplasty where a synthetic graft seeded with MSCs was used ^24^.

The main problems with the clinical application of stem cells in this context are related to the *in vivo* fate of the injected stem cells. Although MSCs are known to migrate to sites of tissue injury even after IV injection, to date there is no clinical evidence to suggest whether these cells engraft successfully after transurethral injection, how long they survive, and whether they can migrate to other body parts. Additionally, the best source of MSCs (autologous versus allogeneic and fat versus bone marrow) and the optimum number of cells needed to achieve a desired effect are not yet known ^19^.

## Tissue engineering-based regenerative therapies

The overall strategy of tissue engineering approaches is to combine a scaffold made of synthetic or natural biomaterials with cells isolated from patients whilst incorporating bioactive factors into these constructs to trigger the desired physiological response. Constructing complex tissues and solid organs is an inherently difficult task, primarily because vascularisation cannot be created and functioning innervation cannot be replicated. Nevertheless, several pilot clinical studies have been conducted showing clinically beneficial outcomes with the use of tissue-engineered oral mucosa ^19^.

Recently, a pilot clinical study investigated another novel tissue engineering approach in six men ^25^. Epithelial cells isolated from patients’ own oral mucosa were encapsulated in a thermoresponsive polymer, which was then injected into the urethra after DVIU. The cell–polymer mixture was also shown to organise into a composite cell-scaffold complex to support the growth of the epithelial cells. Compared to cell-based adjuvant therapies after DVIU, tissue engineering approaches could be advantageous because cell-scaffold complexes can result in the concentration of therapeutic products at the injection site. However, these products require a specialised regulatory pathway before being introduced into mainstream clinical practice.

Recently, tissue-engineered products have been used as an adjunct to DVIU. In a pre-clinical study in rabbits, minced buccal mucosa suspended in fibrin gel, liquid buccal mucosa graft (LBMG), was applied to the site of urethrotomy after DVIU ^26^. The LBMG was shown to effectively graft onto the wound site in 67% of animals, with an improved rate of stricture resolution in the treatment group. This is a proof-of-concept study, and this approach has not been tested in clinical practice.

More extensive reconstruction of the urethra using tissue-engineered urethral substitutes has been ongoing over the past 10 years. The recent pre-clinical and clinical studies investigating a tissue-engineered product for urethroplasty have been summarised in detail elsewhere ^27^. The first use of tissue-engineered oral mucosa for urethral reconstruction was performed by our group in 2008 ^28^. In this study, five patients with lengthy strictures due to lichen sclerosis received a tissue-engineered urethral substitute implantation that was constructed using patients’ own oral mucosa cells. In long-term follow-up, four out of five patients still had their tissue-engineered urethras in place, which looked normal on endoscopic examination ^29^. The first nationally authorised tissue-engineered product, currently legally marketed in Germany, consists of autologous oral mucosa cells seeded on a degradable scaffold. At two-year follow-up, the failure rate with this product was 40% ^30^, with significant variation in success rates between centres involved in the clinical trial. Larger prospective clinical trials with long-term follow-up using standardised outcome assessment measures are needed.

To summarise, tissue engineering offers exciting developments in the management of US disease. There appears to be some progress in constructing a safe and reliable tissue-engineered graft for urethroplasty; however, a better understanding of the mechanisms and risk factors for graft failure with different graft materials and designs is needed. Recent developments in the regulatory processes that need to be followed with the introduction of a tissue engineering approach are discussed in the next section. A further caveat is that such therapies are likely to be associated with high costs as compared to existing surgery using autologous tissue, such as oral mucosa, when introduced into more widespread clinical use.

## Recent changes in the regulatory processes

Advances in biomedical science and technologies over the past few decades have made it more difficult for regulatory bodies to ensure safe processes governing the production, distribution, and approval of advanced therapy medicinal products (ATMPs). Until recently, new treatments coming into clinical practice were regulated under either novel drug treatments or medical devices. These failed to address the complexity and diversity of the newly emerging regenerative medicine products such as cell therapies, tissue engineering, and gene therapies. Here we will briefly summarise the recent changes made to these regulatory processes.

In the EU, new regulations governing the safe use of medical devices entered into force recently ^31^. The regulatory definition of regenerative medicine involves “methods to replace or regenerate human cells, tissues, or organs in order to restore or establish normal function which includes cell therapies, tissue engineering, gene therapy, and biomedical engineering techniques as well as more traditional treatments involving pharmaceuticals, biologics, and devices”. On the other hand, the definition of a tissue-engineered product is much more complicated because of the diversity of its components, which is now subject to regulations under specific legislations ^32^.

Another specific term used by the authorities is ATMP, which refers to a tissue-engineered product that “contains or consists of engineered cells or tissues, and which is presented as having properties for, or is used in, or administered to, human beings with a view to regenerating, repairing, or replacing human tissue ^33^”. An ‘engineered cell’ is defined as “a cell that has been subject to substantial manipulation (such as cutting, grinding, shaping, centrifugation or soaking in antibiotic or antimicrobial solutions) so that biological characteristics, physiological functions or structural properties relevant for the intended regeneration, repair or replacement are achieved”. Additionally, a cell is classified as an engineered cell when it is “not intended to be used for the same essential function or functions in the recipient as in the donor”, for example when an adipose tissue cell is used to regenerate muscle tissue.

Therefore, the above-described tissue-engineered injectable products designed as adjuncts to DVIU and the tissue-engineered urethral substitutes would need to be considered as ATMPs. The regulatory evaluation of ATMPs often requires very specific expertise covering the areas of biotechnology and medical devices. The cellular injectables could also be considered ATMPs depending on the cellular elements involved and how much they have been manipulated.

## Conclusions

Innovative research is being undertaken to achieve better clinical results in the treatment of US disease. The development of tissue-engineered urethral substitutes has received a lot of attention over the past decade. Although this is still ongoing, current research appears to focus on improving the success of DVIU using regenerative medicine and tissue-engineered products. This is a newly developing area, and currently there is a lack of robust evidence for clinical efficacy alongside a lack of standardisation for definitions and unclear regulatory pathways for commercialisation.
